# Investigation of liver diseases using liver Doppler ultrasound in patients with inflammatory bowel diseases

**DOI:** 10.1590/1414-431X2025e14523

**Published:** 2025-10-17

**Authors:** D.N. Shintaku, M.A. Lopes, R.F. Beraldo, E.C.S. de Oliveira, G.S.P. Herrerias, A.C.B. de Oliveira, J.P. Baima, W.F. Barbosa, G.F. Silva, L.Y. Sassaki

**Affiliations:** 1 Departamento de Clínica Médica, Faculdade de Medicina, Universidade Estadual Paulista, Botucatu, SP, Brasil; 2 Fundação Dracenense de Educação e Cultura, Dracena, SP, Brasil

**Keywords:** Liver disease, Nonalcoholic fatty liver disease, Liver ultrasound, Crohn's disease, Ulcerative colitis, Inflammatory bowel disease

## Abstract

Inflammatory bowel disease (IBD), encompassing Crohn's disease (CD) and ulcerative colitis (UC), is characterized by chronic inflammation, which may be associated with hepatic and biliary manifestations such as non-alcoholic fatty liver disease (NAFLD). Despite the high risk of hepatic manifestations among patients with IBD, few studies in Brazil have assessed the frequency of these diseases. Therefore, this study aimed to analyze the prevalence of liver disease by ultrasound in patients with IBD. This was a single-center, cross-sectional study that included patients with IBD who were followed up at an outpatient clinic. The clinical and sociodemographic data, disease activity, biochemical test results, and Doppler liver ultrasonography results were assessed. Descriptive and association tests were used for statistical analyses. A total of 138 patients were included: 64.49% females, mean age 45.55±14.17 years, and body mass index of 26.92±5.07 kg/m^2^. In total, 63 (45.65%) patients had CD and 75 (54.35%) had UC. Most patients were in either clinical (58.39%) or endoscopic remission (52.55%). Liver ultrasound revealed NAFLD in 58 patients (42.03%), which was classified as mild (36.21%), moderate (46.55%), or severe (17.24%). Seven patients had choledocholithiasis and three had chronic liver disease. Liver disease was associated with changes in aspartate aminotransferase (AST), alanine aminotransferase (ALT), hematocrit, hemoglobin, and fasting glucose levels. Liver disease is frequent in IBD patients, with NAFLD being the most prevalent. Screening for liver disease in patients with IBD is recommended for early detection and immediate treatment of the alterations, in order to prevent complications and progression to cirrhosis.

## Introduction

Inflammatory bowel disease (IBD) is an immune-mediated disease characterized by chronic noninfectious inflammation in the intestine. The etiology is multifactorial involving immunological, environmental, and genetic factors. IBD affects a significant portion of the world's population and primarily includes Crohn's disease (CD) and ulcerative colitis (UC) (1).

Patients with IBD are at a greater risk of developing liver disease than the general population because of factors such as persistent systemic inflammation, changes in intestinal permeability with bacterial translocation, increased entry of lipopolysaccharides, free fatty acids, and other toxins directly into the liver through the portal vein, alteration of the intestinal microbiome, use of immunosuppressive medications, and malabsorption of bile salts (2,3). Studies indicate that approximately 0.5 to 5% of patients with IBD develop chronic liver disease (4-6).

Nonalcoholic fatty liver disease (NAFLD) is the most prevalent hepatic manifestation in patients with IBD. A meta-analysis revealed that one-third of all patients with IBD have NAFLD, which is twice as high as that in healthy controls (7). In addition to NAFLD, patients with IBD may present with other hepatic manifestations such as primary sclerosing cholangitis (2-8% prevalence), autoimmune hepatitis (<0.5% prevalence), and choledocholithiasis (8).

Despite the high risk of liver manifestations in patients with IBD and the unfavorable evolution of complications, such as liver cirrhosis and hepatocellular carcinoma, few studies in Brazil have evaluated the frequency of these diseases in this population (9). Investigation of liver disease through ultrasound is essential in these individuals to avoid serious complications. Therefore, the present study aimed to analyze the prevalence of liver changes assessed by Doppler ultrasound in patients with IBD followed up at a reference service to actively seek early detection of changes and the immediate implementation of therapeutic measures indicated for each case. The secondary objective was to identify the main clinical factors associated with the occurrence of liver disease.

## Material and Methods

### Patients and study design

This single-center, cross-sectional study included patients followed up at the IBD Outpatient Clinic of a tertiary hospital (Hospital das Clínicas de Botucatu). Patients of both sexes aged 18 years or older diagnosed with CD or UC confirmed by clinical, laboratory, histological, and/or imaging aspects (1) and diagnosed at least 6 months prior were included. Patients were excluded if they had undetermined colitis, lacked a confirmed diagnosis of IBD, had other chronic inflammatory diseases, such as rheumatoid arthritis and lupus, HIV, or a previous diagnosis of liver disease, such as viral hepatitis, autoimmune hepatitis, primary sclerosing cholangitis, primary biliary cirrhosis, overlap syndrome, storage diseases, or other chronic liver diseases. Additionally, patients using prednisone at a dose >20 mg/day and those with alcohol consumption >30 g/day for men and >20 g/day for women were excluded.

This study was approved by the hospital's Research Ethics Committee (CAAE: 42073421.7.0000.5411). All participants received an explanation about the study aims and expected results and were only enrolled in the study after signing an informed consent form. Participants' anonymity was guaranteed.

### Sociodemographic and clinical variables

Sociodemographic variables, such as age, sex, family income, ethnicity, alcohol consumption, smoking, body mass index (BMI), presence of comorbidities, medications used, type of IBD, activity and extent of the disease, previous surgery for IBD, and presence of an ostomy were evaluated. We also evaluated the use of medications for the treatment of IBD such as 5-aminosalicylic acid derivatives, immunosuppressants such as azathioprine and JAK inhibitors, and biological therapies such as infliximab, adalimumab, golimumab, certolizumab pegol, vedolizumab, and ustekinumab.

### Classification and assessment of the inflammatory activity of IBD

CD was classified using the Montreal Classification (10). Disease activity classification was based on the Crohn's Disease Activity Index (CDAI) (11). The patients were classified as having clinical remission or mild, moderate, or severe clinical activity. The extent of UC was classified into distal colitis, pancolitis, and left colitis (12). Disease activity was determined using the Mayo Score (13).

### Biochemical tests

Biochemical tests, including blood count, fasting blood glucose, glycated hemoglobin, basal insulin, lipid profile, tests of inflammatory activity such as C-reactive protein and erythrocyte sedimentation rate (ESR), and liver tests such as gamma-glutamyl transferase (GGT), aspartate aminotransferase (AST), and alanine aminotransferase (ALT) were evaluated.

### Liver ultrasound

Ultrasound examinations were performed by a single examiner using a Saevo model FT412 device (Saevo, Brazil). Liver ultrasound was used to assess the presence of NAFLD, chronic liver disease, gallstone disease, and hepatic thromboembolism. The diagnosis of NAFLD by ultrasonography has a sensitivity of 82-89% and specificity of 93% (14) and can be classified as Grade I (mild): increased echogenicity or brightness of the liver with normal visualization of the diaphragm and edges of the intrahepatic vessels; Grade II (moderate): increased echogenicity with slight attenuation of the posterior beam, resulting in difficulty in visualizing the diaphragm and intrahepatic vessels; and Grade III (intense): a significant increase in echogenicity and marked attenuation of the posterior beam makes it impossible to visualize the diaphragm and intrahepatic vessels of the right lobe of the liver (15).

### Statistical analysis

Descriptive analysis was carried out to characterize the population by calculating the mean and standard deviation or median and interquartile range (25th-75th) for quantitative variables and frequencies and proportions for qualitative variables. For comparison between groups and analysis of variables associated with detection of liver disease in the ultrasound, the chi-squared test and Fisher's exact test were used for categorical variables and the Student's *t*-test was used for continuous variables. The Kruskal-Wallis test was used to compare non-parametric variables. Statistical significance was set at P<0.05.

## Results

Initially, 168 patients were recruited, but 30 were excluded since they did not undergo liver ultrasonography; therefore, the study sample consisted of 138 patients. The average age was 45.55±14.17 years. There was a higher prevalence of females (64.49%), patients who were active in a profession (53.62%), had a family income of 2 to 3 minimum wages (39.42%), and had completed high school education (40.15%). Low frequencies of social drinking (27.54%) and smoking (6.52%) were observed (Table 1).

**Table 1:** Sociodemographic characteristics of patients with IBD based on the presence or absence of liver diseases visualized on ultrasound.

Characteristics	IBD(n=138)	Patients with liver disease(n=61)	Patients without liver disease (n=77)	P
Age (years)	45.55±14.17	48.38±12.52	43.29±15.07	**0.0363**
Female	89 (64.49)	33 (54.10)	56 (72.73)	**0.0231**
Marital status				0.1179
Stable union	78 (56.52)	39 (63.93)	39 (50.65)	
Others	60 (43.48)	22 (36.07)	38 (49.35)	
Educational level				0.5918
Primary school	45 (32.85)	18 (30.00)	27 (35.06)	
Secondary school	55 (40.15)	27 (45.00)	28 (36.36)	
Higher education	37 (27.01)	15 (25.00)	22 (28.57)	
Caucasian ethnicity	101 (73.19)	45 (73.77)	56 (72.73)	0.8907
Active in profession	74 (53.62)	36 (59.02)	38 (49.35)	0.2581
Family income				0.2491
Less than 1 minimum wage	1 (0.96)	0	3 (3.90)	
1-2 minimum wages	33 (31.73)	19 (31.67)	29 (37.66)	
2-3 minimum wages	41 (39.42)	26 (43.33)	24 (31.17)	
3 or more minimum wages	29 (27.88)	15 (25.00)	21 (27.27)	
Social alcohol intake	38 (27.54)	21 (34.43)	17 (22.08)	0.1068
Smoking	9 (6.52)	4 (6.56)	5 (6.49)	0.9880
Comorbidities				
Diabetes	14 (10.14)	8 (13.11)	6 (7.79)	0.3037
Hypertension	32 (23.19)	20 (32.79)	12 (15.58)	**0.0174**
Dyslipidemia	16 (11.59)	11 (18.03)	5 (6.4	**0.0355**
Hypothyroidism	9 (6.52)	3 (4.92)	6 (7.79)	0.4971
Hyperthyroidism	1 (1.30)	0	1 (1.30)	0.3717
Autoimmune disease	26 (20.97)	13 (23.64)	13 (18.84)	0.5146
BMI (kg/m^2^)	26.92±5.07	29.56±4.19	24.81±4.72	**<0.0001**
BMI classification				**<0.0001**
Malnourished	3 (2.19)	0	3 (3.95)	
Eutrophic	44 (32.12)	6 (9.84)	38 (50.00)	
Overweight	58 (42.34)	32 (52.46)	26 (34.21)	
Obesity	32 (23.36)	23 (37.70)	9 (11.84)	

Data are reported as means±SD or number (percentage). P-values in bold are statistically significant. Chi-squared test, Fisher's exact test, or Student's *t*-test. IBD: inflammatory bowel disease; BMI: body mass index.

Of the 138 patients who underwent liver ultrasound, 61 had liver disease, most of whom had NAFLD (n=58, 42.03%). Among patients with NAFLD, 26 (41.27%) had UC and 32 (42.67%) had CD. NAFLD was classified as mild (36.21%), moderate (46.55%), and severe (17.24%). Seven (3.84%) cases of cholelithiasis were identified, and three (2.17%) patients had chronic liver disease. No patient had portal, superior mesenteric, or splenic vein thrombosis (Table 1).

There was a lower proportion of women (P=0.0231) among patients with liver disease, who were also older (P=0.0363). Hypertension (P=0.0174) and dyslipidemia (P=0.0355) were more frequent in the group with liver abnormalities, as was the frequency of being overweight and obese according to BMI classification (Table 1), compatible with the prevalence of NAFLD.

**Figure 1:**
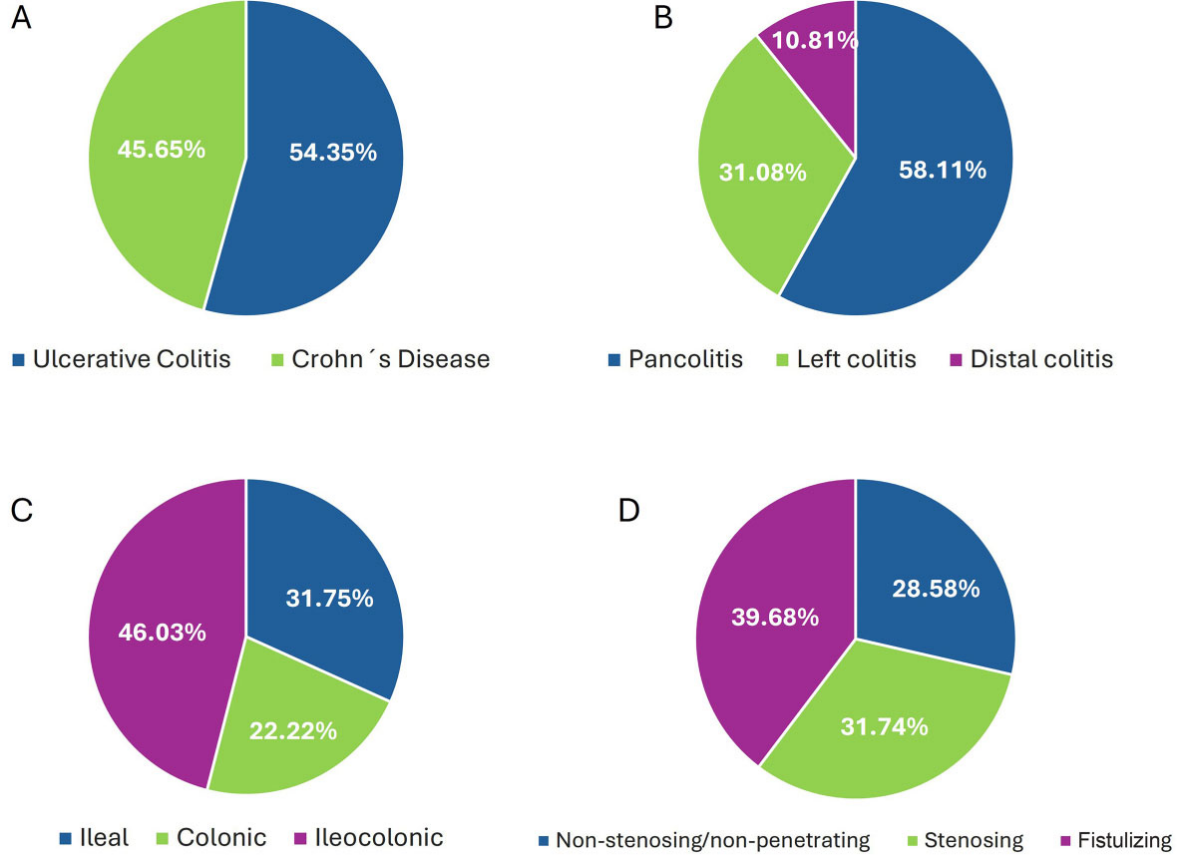
Characterization of patients with inflammatory bowel disease (IBD). **A**, Distribution of patients according to disease type (Crohn's disease (CD) or ulcerative colitis (UC)). **B**, Distribution of patients with ulcerative colitis according to disease extent. **C**, Distribution of patients with Crohn's disease according to disease extent. **D**, Distribution of patients with Crohn's disease according to disease behavior.

Regarding the clinical characteristics of IBD in the total sample, the mean time since diagnosis was 10 (6-16) years. Seventy-five patients had UC (54.35%) and 63 had CD (45.65%) (Figure 1). Among the patients with CD, the disease extent was ileocolonic in 46.03%, ileal in 31.75%, and colonic in 22.22%. Disease behaviors were non-stenosing/non-penetrating in 28.58%, stenosing (31.74%), and fistulizing in 39.68%; perianal manifestations were present in 35.48% (Figure 1).

Among patients with UC, the extensive disease predominated (58.11%), followed by left colitis (31.08%), and distal colitis (10.81%) (Figure 1). The presence of extraintestinal manifestations (EIM) was common (74.77%), and the most frequent manifestations were arthralgia (38.53%), oral ulcers (12.84), and uveitis (8.26%). Overall, clinical activity was observed in 57 patients (41.61%), and endoscopic activity was observed in 65 patients (47.45%). No association was detected between disease activity and presence of liver disease. However, an association was observed between a longer time since diagnosis and the presence of liver abnormalities (P=0.0497) (Table 2).

**Table 2:** Clinical characteristics of patients with IBD based on the presence or absence of liver disease visualized on liver ultrasound.

Characteristics	IBD(n=138)	Patients with liver disease(n=61)	Patients without liver disease (n=77)	P
IBD classification				
Crohn's disease	63 (45.65)	27 (44.26)	36 (46.75)	0.7705
Ulcerative colitis	75 (54.35)	34 (55.74)	41 (53.25)	
Disease duration (years)	10.0 (6.0-16.0)	12.0 (6.0-18.50)	9.0 (5.0-15.0)	**0.0497**
HBI score	3.0 (2.0-5.0)	2.0 (2.0-5.0)	3.0 (2.0-5.0)	0.3939
CDAI score	78.0 (38.0-138.0)	78.0 (34.0-104.0)	78.0 (39.0-151.0)	0.3815
CD activity according to CDAI				
Remission	49 (77.78)	23 (85.19)	26 (72.22)	0.1195
Mild	10 (15.87)	4 (14.81)	6 (16.67)	
Moderate	4 (6.35)	0	4 (11.11)	
Severe	0	0	0	
Total Mayo score	2.0 (0-4.0)	2.0 (1.0-3.0)	2.0 (0 -4.0)	1.0
UC activity according to Mayo				
Remission	31 (41.89)	14 (42.42)	17 (41.46)	0.6889
Mild	29 (39.19)	12 (36.36)	17 (41.46)	
Moderate	8 (10.81)	3 (9.09)	5 (12.20)	
Severe	6 (8.11)	4 (12.12)	2 (4.88)	
Clinical active disease	57 (41.61)	23 (38.33)	34 (44.16)	0.4927
Endoscopic active disease	65 (47.45)	30 (50.00)	35 (45.45)	0.5971
Presence of EIM	83 (74.77)	39 (78.00)	44 (72.13)	0.4787
Arthralgia	42 (38.53)	21 (43.75)	21 (34.43)	0.3207
Uveitis	9 (8.26)	4 (8.33)	5 (8.20)	0.9795
Erythema nodosum	6 (5.50)	1 (2.08)	5 (8.20)	0.1648
Pyoderma	3 (2.75)	1 (2.08)	2 (3.28)	0.7049
Oral ulcer	14 (12.84)	4 (8.33)	10 (16.39)	0.2118
Previous surgery				
Perianal	14 (10.14%)	5 (8.20)	9 (11.69)	0.4999
Enterectomy	23 (16.67%)	11 (18.03)	12 (15.58)	0.7015
Ostomy	3 (2.17%)	1 (1.64)	2 (2.60)	0.7015

Data are reported as means±SD, median and interquartile range (25th-75th), or number (percentage). P-values in bold are statistically significant. Chi-squared test, Fisher's exact test, Student's *t*-test, or Kruskal-Walli's test. IBD: inflammatory bowel disease; HBI: Harvey-Bradshaw index; CDAI: Crohn's disease activity index; CD; Crohn's disease; UC: ulcerative colitis; EIM: extraintestinal manifestations.

**Figure 2:**
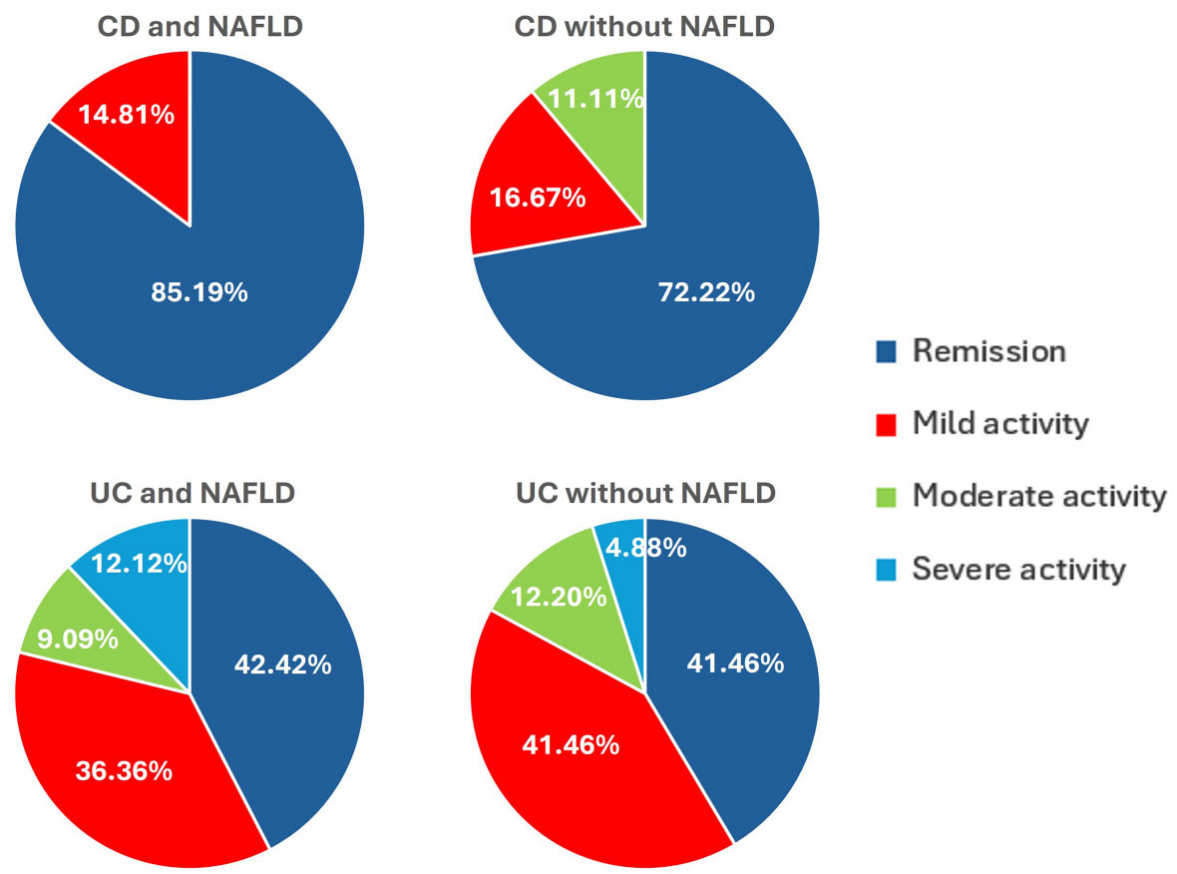
Classification of disease activity according to the presence or absence of non-alcoholic fatty liver disease (NAFLD) in patients with Crohn's disease (CD) and ulcerative colitis (UC).

The clinical activity of IBD differed between patients with CD and UC. The majority of patients with CD and NAFLD were in clinical remission (85.19%), whereas in the group with CD without NAFLD, 72.22% were in clinical remission, 16.67% in mild activity, and 11.11% in moderate activity. Among the patients with UC and NAFLD, 42.42% were in remission and 36.36% in mild, 9.09% in moderate, and 12.12% in severe activity. Of the patients with UC without NAFLD, 41.46% were in remission and 41.46% in mild, 12.20% in moderate, and 4.88% in severe activity (Figure 2).

Biological therapy and azathioprine were used to treat IBD in 45.65 and 42.75% of patients, respectively. Prednisone at a dose <20 mg/day was used in 13.77% of the patients. The use of budesonide, although at a low rate, was more frequent in patients with liver abnormalities (P=0.0359) (Table 3).

**Table 3:** Medications used by patients with IBD and based on the presence or absence of liver disease visualized on liver ultrasound.

Medications	IBD(n=138)	Patients with liver disease(n=61)	Patients without liver disease (n=77)	P
Prednisone	19 (13.77)	8 (13.11)	11 (14.29)	0.8428
Budesonide	9 (6.52)	7 (11.48)	2 (2.60)	**0.0359**
Azathioprine	59 (42.75)	28 (45.90)	31 (40.26)	0.5058
Infliximab	31 (22.46)	13 (21.31)	18 (23.38)	0.7728
Adalimumab	25 (18.12)	11 (18.03)	14 (18.18)	0.9820
Antibiotics	3 (2.17)	1 (1.64)	2 (2.60)	0.7015
Biological therapy	63 (45.65)	27 (44.26)	36 (46.75)	0.7705
Previous exposure to biological therapy	37 (26.81)	16 (26.23)	21 (27.27)	0.8907

Data are reported as number (percentage). P-values in bold are statistically significant (chi-squared test). IBD: inflammatory bowel disease; US: ultrasound.

In the evaluation of biochemical tests, patients with liver changes had significantly higher values of fasting blood glucose, basal insulin, triglycerides, and liver biochemicals such as AST, ALT, ALP, and gamma GT (Table 4).

**Table 4:** Biochemical characteristics of patients with IBD and based on the presence or absence of liver disease visualized on liver ultrasound (US).

Characteristics	IBD(n=138)	Patients with liver disease on liver US (n=61)	Patients without liver disease on liver US (n=77)	P
Hematocrit (%)	42.00±4.31	43.47±4.42	40.86±3.87	**0.0003**
Hemoglobin (g/dL)	13.98±1.56	14.53±1.61	13.55±1.39	**0.0002**
Platelets (mm^3^)	268.000 (225.000-324.000)	263.500 (225.000-309.000)	276.000 (221.000-342.000)	**<0.0001**
Leukocytes (mm^3^)	6800 (5400-8800)	6850 (5550-8650)	6800 (5400-9000)	**<0.0001**
Fasting blood glucose (mg/dL)	88.0 (84.0-99.0)	95.0 (86.0-108.0)	86.0 (81.0-92.50)	**0.0003**
Glycated hemoglobin (%)	5.40 (5.20-5.90)	5.50 (5.10-6.0)	5.30 (5.20-5.70)	0.1628
Triglycerides (mg/dL)	137.0 (92.0-195.0)	153.0 (102.0-210.0)	104.0 (80.0-146.0)	**0.0022**
LDL (mg/dL)	129.40±29.94	133.50±7.78	127.35±38.17	0.8415
HDL (mg/dL)	53.79±15.50	48.75±15.28	58.20±14.47	**0.0075**
Total cholesterol (mg/dL)	191.10±42.44	193.23±47.38	188.89±36.95	0.6083
Insulin (micro-IU/mL)	8.35 (5.50-13.40)	11.0 (8.0-19.90)	6.0 (4.70-9.50)	**<0.0001**
Ferritin (ng/dL)	63.90 (20.70-144.0)	103.0 (28.70-226.40)	33.10 (12.9-74.30)	**0.0024**
Creatinine (mg/dL)	0.7 (0.6-0.9)	0.8 (0.7-0.9)	0.7 (0.6-0.9)	0.0528
Albumin (g/dL)	4.30 (4.0-4.5)	4.20 (4.0-4.4)	4.30 (4.0-4.5)	0.4693
C-reactive protein (mg/dL)	0.50 (0.50-1.60)	0.50 (0.50-1.50)	0.70 (0.50-1.6)	0.3111
Erythrocyte sedimentation rate (mm/h)	13.0 (7.0-27.0)	10.5 (6.0-25.0)	13.0 (7.0-27.0)	0.7303
AST (U/L)	24.0 (20.0-29.0)	25.0 (22.0-29.0)	23.0 (19.0-27.0)	**0.0435**
ALT (U/L)	19.50 (14.0-26.0)	23.0 (17.0-33.0)	17.0 (12.5-23.0)	**<0.0001**
GGT (U/L)	26.50 (19.0-41.0)	31.0 (25.0-45.0)	23.0 (17.0-37.0)	**0.0003**
ALP (U/L)	66.0 (53.0-80.0)	63.0 (53.0-80.0)	67.0 (50.0-81.0)	**0.0001**
Total bilirubin (mg/dL)	0.70 (0.60-0.90)	0.70 (0.60-0.90)	0.70 (0.60-0.90)	0.6984

Data are reported as means±SD or median and interquartile range (25th-75th). P-values in bold are statistically significant. Student's *t*-test and Kruskal-Walli's test. IBD: inflammatory bowel disease; LDL: low-density lipoprotein; HDL: high-density lipoprotein; AST: aspartate aminotransferase; ALT: alanine aminotransferase; GGT: gamma-glutamyl transferase; ALP: alkaline phosphatase.

## Discussion

The link between IBD and NAFLD has gained traction in the literature. Several studies demonstrate that patients with IBD have an increased risk of developing NAFLD (16-18). This association can be explained by certain factors such as chronic inflammation and dietary changes. IBD is characterized by persistent inflammation of the digestive tract, leading to the production of substances that can cause liver damage and contribute to fat accumulation. Patients with IBD may have difficulty absorbing nutrients, leading to malnutrition and vitamin deficiency. Certain dietary changes such as reduced fiber intake may also increase the risk of NAFLD. Other contributing factors include metabolic syndrome, genetic predisposition, altered immune response, use of medications, and changes in the microbiota with an increased uptake of endotoxins from the intestine by the liver (19,20).

Primary sclerosing cholangitis, cholelithiasis, fatty liver disease, hepatic amyloidosis, granulomatous hepatitis, drug-induced liver injury, venous thromboembolism, primary biliary cholangitis, IgG4-related cholangiopathy, autoimmune hepatitis, and liver abscesses or reactivation of viral hepatitis are among the liver diseases associated with IBD (21).

In the present study, a high percentage of patients who underwent liver ultrasound examinations presented with NAFLD (42.03%). This contrasts with the findings of a study conducted in São Paulo (22) that evaluated the abdominal ultrasound of 9,340 individuals from the general population and found a 19.2% prevalence of NAFLD. This shows that patients with IBD have a higher prevalence of hepatic steatosis than the general population, which is in line with previous studies that also showed a higher rate of NAFLD in patients with IBD, with a prevalence ranging from 33 to 55% (16-18).

A national study in Brazil analyzed the clinical and histological profiles of steatosis in 1280 patients diagnosed with NAFLD and revealed that 42% of the patients had isolated hepatic steatosis, and 27, 15.4, and 0.7% had fibrosis, cirrhosis, and hepatocellular carcinoma, respectively (23).

NAFLD is a chronic condition characterized by triglyceride accumulation (steatosis) in more than 5% of hepatocytes resulting from an imbalance in lipid metabolism (18). It can range from mild steatosis to non-alcoholic steatohepatitis, which can progress to liver cirrhosis and even hepatocellular carcinoma (24). Studies have shown a correlation between patients with NAFLD and IBD (18,25). In patients with IBD, NAFLD is probably due to persistent inflammation and the continuous use of corticosteroids and immunosuppressants (18). Furthermore, inflammation caused by IBD can alter the intestinal microbiome of patients, resulting in the modification of lipid metabolism and consequently leading to NAFLD (25). The main pathophysiological mechanisms that explain this high prevalence are recurrent chronic inflammation, changes in intestinal microbiota, parenteral nutrition, potentially hepatotoxic medications, and surgery (21). Other risk factors for NAFLD include metabolic syndrome, obesity, and diabetes (24). Since patients with IBD are at an increased risk of metabolic syndrome due to the presence of chronic inflammation, they are probably at a greater risk for the development of NAFLD. Early detection of this disease is important for immediate treatment and control of the risk factors, thus reducing the risk of progression to cirrhosis, liver fibrosis, and other complications.

Recently, the name NAFLD was revised and a new nomenclature, metabolic dysfunction-associated steatotic liver disease with (MASLD), was adopted (26). This condition ranges from fat deposition in hepatocytes characterized by ≥5% hepatic steatosis to steatohepatitis associated with metabolic dysfunction (MASH) and advanced stages of the disease, such as advanced fibrosis, liver cirrhosis and complications including hepatocellular carcinoma (27). The diagnosis of MASLD includes the presence of hepatic steatosis, and at least one of the following five cardiometabolic criteria: BMI compatible with overweight/obesity or increased waist circumference, altered blood glucose or type 2 diabetes, arterial hypertension, change in triglyceride levels or treatment with lipid-lowering drugs, and change in HDL levels or lipid-lowering treatment (26,28). In the present study, the term NAFLD was maintained, as we assessed the presence of hepatic steatosis on liver ultrasound examination and did not classify patients as MASLD.

A Brazilian article recently published by Oliveira et al. (29) presented data on MASLD in patients with IBD followed at one reference center. The percentage of MASLD (44.3%) was very similar to that found in our study (42.03%), and the factors associated with the presence of MASLD were older age and factors associated with metabolic syndrome, as well as a longer time since diagnosis of IBD, as in the present study, showing similar data in the two Brazilian centers. A higher frequency of MAFLD was also observed in a Spanish study that included 830 IBD patients and 1718 controls. The prevalence of MAFLD and advanced liver fibrosis was 42 and 9.5% in IBD patients and 32.77 and 2.31% in the general population (P<0.001) (30).

Cholelithiasis occurs when stones form in the gallbladder due to different factors, such as cholesterol supersaturation, hypomotility, a defect in the gallbladder, or excess bilirubin. Gallstones can have different compositions according to their etiology and can cause various complications such as cholecystitis, choledocholithiasis, pancreatitis, and cholangitis (31). Studies have indicated that CD is related to the formation of gallbladder stones, particularly when the ileum is affected (32,33). CD causes changes in the bile composition, which becomes supersaturated, with an increase in the bilirubin concentration and a decrease in the percentage of deoxycholic acid, making individuals with IBD susceptible to gallbladder stone formation (34).

The prevalence of cholelithiasis in the general population ranges from 5.5 to 15% (35). In CD, the risk of developing gallstones is higher (prevalence rate 11-34%), whereas no differences in prevalence compared with the general population has been identified in UC (21). The main risk factors for cholelithiasis in patients with CD are age at the time of CD diagnosis, disease duration (>15 years), ileocolonic location of the lesions, length of ileal resection (>30 cm), frequency of disease recurrence (>3), prolonged hospital stay, greater number of hospitalizations (>3), and the need for total parenteral nutrition (31). The pathophysiological mechanisms related to cholelithiasis in CD include bile acid malabsorption, alteration of bilirubin solubilization by unabsorbed bile acids in the colon, dysbiosis leading to altered bile acid metabolism, and reduced vesicular motility (21). Another interesting point is that CD and cholelithiasis are more closely related than UC and cholelithiasis because of the decreased absorption of bile salts in the ileum (31,32).

Regarding medications, there is a correlation between budesonide use and liver disease on ultrasound. A meta-analysis reported a link between liver injury and thiopurine treatment, but not with anti-TNFα therapies (36). A study that followed 229 patients with IBD who received azathioprine or 6-mercaptopurine reported the presence of liver damage in 9% of the patients (37). A significant difference in BMI was also identified between patients with and without liver injury (BMI 27.6 *vs* BMI 24.2; P=0.002) (37). Some studies have attempted to correlate the use of glucocorticoids with NAFLD, but these were *in vitro* and animal studies, and the results do not establish the use of these medications as an independent risk factor for hepatic steatosis (20).

Tracking NAFLD using ultrasound is crucial to prevent disease progression to more advanced and potentially fatal stages. It is estimated that 25-30% of patients with NAFLD progress to nonalcoholic steatohepatitis, and approximately 20% of these develop liver cirrhosis, significantly increasing the risk of hepatocellular carcinoma (38). The onset of liver cirrhosis, in turn, results in an exponential increase in morbidity and mortality and a deterioration in the patient's quality of life.

Therefore, early diagnosis of NAFLD is extremely important. Adequate clinical and therapeutic monitoring can help slow the natural progression of the disease, prevent the development of serious complications, and improve the quality of life of patients. Additionally, NAFLD screening should be considered as an essential public health measure that promotes early diagnosis and timely intervention, significantly contributing to the reduction in morbidity, mortality, and disease burden associated with NAFLD.

One of the limitations of the study was the number of participants. There were difficulties in including some patients. For instance, some patients reported transportation difficulties to the clinic for examinations because they lived in other cities, which resulted in the exclusion of 30 patients. However, an important number of patients were included in the present study. Another limitation was the lack of magnetic resonance imaging (MRI). Although MRI is the most accurate examination for detecting hepatic steatosis (39), due to its high cost, it was not possible to perform this examination in our study, and liver ultrasound was chosen. Unfortunately, liver elastography was not available at the service. Although some previous studies excluded metabolic criteria to rule out hepatic steatosis caused by metabolic syndrome, in this study the patient was evaluated in his or her integral condition, considering the associated metabolic comorbidities. Differently, in a retrospective study, the authors evaluated 143 patients with IBD who underwent hepatic ultrasound and divided them into two different groups according to the presence or absence of NAFLD. The prevalence of NAFLD was 23%, and 26/33 patients with NAFLD did not meet criteria for metabolic syndrome (40), which may attribute the presence of NAFLD to other factors associated with systemic chronic inflammation.

In conclusion, IBD can lead to extraintestinal manifestations, including liver damage. The spectrum of associated liver injuries varies and includes cholestatic liver disease, NAFLD, gallstones, and portal vein thrombosis. Careful monitoring of liver function and assessment of the risk of liver damage through laboratory tests such as bilirubin and serum and canalicular aminotransferases should be performed. Subsequent imaging studies, indicated by clinical manifestations and biological changes, should be performed. Early diagnosis of liver disease and initiation of specific therapeutic measures can improve the prognosis of patients with IBD and prevent progression to cirrhosis. Furthermore, patients should be informed about the risk of developing liver diseases and about possible hepatotoxic substances that accelerate the progression to liver disease, such as alcohol, high sugar consumption, and industrialized and ultra-processed foods. A multidisciplinary team is the key to the successful care of these systemic diseases.
